# Longitudinal Association Between Falls and Depressive Symptoms Among Community-Dwelling Older Adults in China

**DOI:** 10.3390/bs16020228

**Published:** 2026-02-04

**Authors:** Meng Jiang, Kuiyu Tang, Yueyun Zhang

**Affiliations:** 1 School of Social Sciences, Harbin Institute of Technology, Harbin 150001, China; tangky@hit.edu.cn; 2 School of Philosophy and Social Development, Shandong University, Jinan 250100, China; zyueyun@sdu.edu.cn

**Keywords:** fall, depression, social adaptation, Hukou, China, older adults

## Abstract

Purpose: Falls and depression in later life are both public health concerns. The current study aimed to establish the longitudinal link between falls and depressive symptoms among community-dwelling older adults in China. Moreover, the potential mediating role of social adaptation and moderating role of hukou (i.e., household registration system) were explored. Methods: Data were from four consecutive waves of a nationwide, longitudinal survey of community-dwelling older adults in China, encompassing a total of 31,526 person-year observations from 11,092 individual older adults. Both falls and depressive symptoms were self-reported. Random effects regression models were used to estimate the longitudinal association between falls and depressive symptoms. Mediation analysis and moderation were further employed to investigate the mediating role of social adaptation and moderating role of hukou, respectively. Results: We observed a positive longitudinal association between falls and depressive symptoms (β = 0.625 *p* < 0.001). Social adaptation played a partial mediating role, accounting for 8.91% (95% CI [0.037, 0.075]) of the association. Compared with their rural counterparts, urban older adults experienced significantly higher effects of falls on depression (β = 0.320, *p* < 0.01). Conclusions: These findings underscore the benefits of fall prevention interventions for elderly depression and advance our understanding of the psychosocial pathways linking falls to psychological outcomes in older adults.

## 1. Introduction

Depression has become a major public health problem worldwide (Rong et al., 2024). According to estimates from the World Health Organization, approximately 280 million people suffer from depression, accounting for 5 percent of adults overall and 5.7 percent of those aged 60 years and older (WHO, 2025). China has the largest elderly population in the world. The prevalence of depression among Chinese older adults is notably severe (Tang et al., 2021), as compared to the middle-aged or younger population (Phillips et al., 2009). A recent estimate showed that the prevalence of depression among Chinese older adults was 23.02% (Cheng et al., 2022). Depressive symptoms can lead to multiple adverse consequences, affecting the quality of life and social functioning of the elderly and can even lead to suicidal ideation and behavior (Abdoli et al., 2021; Curran et al., 2020; J. Jang et al., 2021). Given the high prevalence and negative consequences of depression in later life, it has become a collective imperative to explore the influencing factors and mechanisms of depression in older adults.

Falls, by definition, are typically an event in which a person unintentionally comes to rest on the ground, floor, or a lower level. The occurrence of falls is particularly common among older adults due to declines in body strength and balance. Each year, one-third of adults aged 65 and older and one-half of those aged 80 and older experience at least one fall worldwide (Mattle et al., 2020; Phelan, 2018). China is no exception, with an estimated prevalence rate of falls among the elderly ranging from 18.0% to 19.0% (Kwan et al., 2011; Peng et al., 2019). In terms of consequences, falls have been considered one of the serious health events during older adulthood (Stewart Williams et al., 2015), often triggering substantial physiological, social, and psychological impairments (Rao et al., 2018). Beyond falls-related injuries such as fractures and head trauma (Harada, 2013; Peirce et al., 2000; Saadat et al., 2016), falls are increasingly recognized as a risk factor for adverse psychological outcomes (Hoffman et al., 2017), including heightened loneliness and anxiety (Yu et al., 2025; Yue et al., 2022), for instance. However, the longitudinal association between falls and depressive symptoms in later life remains underexamined in the China context, with particularly little attention to the underlying mechanisms and heterogeneity across population groups.

A growing body of research suggests that falls may contribute to depressive symptoms in older adults, but this relationship remains insufficiently examined relative to the physical consequences of falls. The occurrence of falls often triggers a wide range of consequences. Studies have shown that falls are associated with functional limitations, reduced self-efficacy and increased anxiety (Y. Jang et al., 2025; Lo et al., 2014; Wang et al., 2010; Yue et al., 2022), all of which are key contributors to the onset and progression of depression. In particular, the experience of falls can lead to a loss of independence and an aggravation of social isolation (Kvelde et al., 2010; Rao et al., 2018), placing older adults at greater risk for mental health issues. From a theoretical perspective, this process can be understood through the fear-avoidance model, which posits that a fall may evoke fear of recurrent falls, leading individuals to avoid social activities. Over time, such avoidance behaviors may contribute to social withdrawal and deteriorating mental health (Peeters et al., 2020). Although some studies have examined cross-sectional associations between falls and psychological symptoms such as loneliness and anxiety, and several longitudinal studies have assessed the relationship between falls and depressive symptoms among older adults in China, relatively limited attention has been paid to the mechanisms through which falls influence depression and the heterogeneity of the population. Further clarification of these pathways is important for understanding post-fall psychological trajectories and for informing targeted interventions.

**Figure 1:**
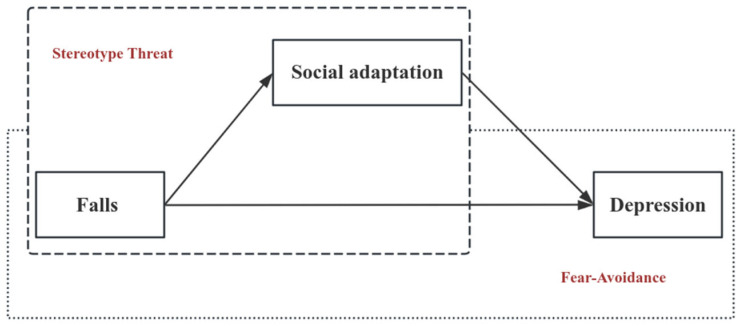
Conceptual model.

Extending prior research, this study additionally examined whether and how social adaptation can mediate the association between falls and depressive symptoms among older adults. Social adaptation represents an important psychosocial concept, referring to the “process of maintaining a positive mindset, accepting the realities of later life, and continuously making self-adjustments to adapt to the challenges of aging” (W. C. Chen & Wang, 2024; Ye et al., 2022). On the one hand, falls are a powerful negative event in older adults, which may directly impair social adaptation. Drawing on the stereotype threat model (Chasteen & Cary, 2015), older adults who have fallen are more likely to internalize aging-related stereotypes, thereby reducing their social networks and social participation (Ruthig et al., 2007; Yue et al., 2022), and ultimately weakening their social adaptation. Moreover, previous studies have demonstrated a positive link between social adaptation and mental health among Chinese older adults (W. C. Chen & Wang, 2024). Therefore, falls may indirectly increase the risk of depression by reducing social adaptation. The conceptual model of this study is presented in Figure 1.

Based on the China context, this study further explored whether the association between falls and depression may vary by the Hukou status of respondents. Hukou is a persistent and unique household registration system in China, distinguishing the whole population between urban and rural Hukou holders. Compared to rural Hukou, urban Hukou has long been linked to socioeconomic advantages including education opportunities, living conditions, health securities, and other welfare benefits. Therefore, urban and rural older adults may employ different coping mechanisms when faced with falls. After a fall, urban older adults may be more inclined to experience decreased social participation, whereas rural older adults may maintain daily interaction and communication because village space is compact, and blood-kinship networks are visible and accessible (Merril et al., 2006). To the best of our knowledge, limited evidence exists regarding Hukou differences in the association between falls and depression (Wang et al., 2023).

To address these gaps, this study examined the longitudinal association between falls and depression in Chinese older adults, and further explored whether social adaptation can mediate the association and whether Hukou status can play a moderating role. The data used in this study were obtained from the China Longitudinal Aging Social Survey (CLASS), a nationally representative longitudinal study of older adults in China. Four consecutive waves of CLASS (2014, 2016, 2018, and 2020) were included in our analysis. Random-effects regression models were employed by including a person-specific error term to the conventional model, thereby accounting for the correlation across repeated observations within each respondent. We selected random effects over fixed-effects models because this approach allowed us to estimate both within-person temporal changes and between-person differences simultaneously, while efficiently utilizing all available data and maintaining statistical power for time-invariant covariates in our longitudinal analysis. Baron and Kenny method and Bootstrap strategies were used to explore the mediating role of social adaptation in the relationship between falls and depression. Finally, stratified regression and an interaction model were used to explore the moderating effect of Hukou.

## 2. Materials and Methods

### 2.1. Data and Participants

CLASS is a nationwide longitudinal survey of adults aged 60 or older in China. It has been conducted every 2 or 3 years since its baseline survey in 2014. CLASS gathers information on a range of factors, including personal basic information, health and related services, socio-economic status, retirement planning and social support, psychological feelings, family and children, daily activities, and exercise among older adults. Participants in the baseline survey were selected through a multistage stratified sampling process. More detailed information on the sampling design and data collection procedures is available at http://class.ruc.edu.cn. At the time the current study was initiated, four waves of data from CLASS (2014, 2016, 2018, 2020) were available for public application. Therefore, this study utilized longitudinal data that were collected over a 6-year period, from Wave 1 in 2014 to Wave 4 in 2020. The initial observations collected were 11,511 in wave 1, 11,471 in wave 2, 11,419 in wave 3, and 11,398 in wave 4, respectively.

**Figure 2:**
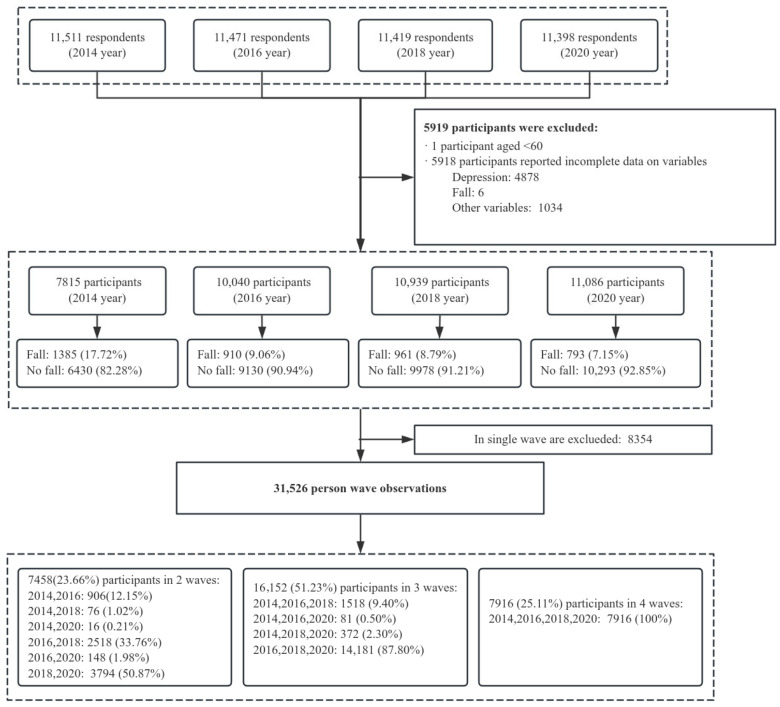
Flowchart of sample selection.

We followed several steps to obtain our final analytical sample. Eligible participants were community-dwelling older adults aged 60 years and above who provided valid information on falls, depressive symptoms, and key covariates across at least two survey waves. First, we excluded 4878 observations with missing values on key independent variables and 1 observation aged less than 60 years. Second, we further dropped 6 observations due to missing values on falls and 1034 observations due to missing values on control variables. Third, to serve our longitudinal research design, we additionally excluded 8354 single-wave observations. As a result, a total of 31,526 observations for 11,092 older adults were used for analysis. The sample selection procedure for this study is summarized in Figure 2.

### 2.2. Measures

#### 2.2.1. Outcome Variable

Depressive symptoms were measured using the brief version of the Center for Epidemiological Studies-Depression (CES-D9) (Radloff, 1977). The CES-D9 scale comprised nine items designed to assess the frequency of specific emotional and behavioral manifestations experienced by respondents within the past week, including items such as “felt pleasant”, “felt lonely”, and “slept poorly”. The responses ranged from 0 (none) to 2 (often). Cronbach’s alpha coefficients for the items at T1 were 0.932, indicating high internal consistency. After reverse coding the reverse questions, the scores of all 9 items were aggregated. A higher overall score indicates a greater degree of depressive symptoms.

#### 2.2.2. Key Independent Variable

Falls were evaluated using a single-item measure derived from the following question: “In the past twelve months, have you ever fallen?” Respondents could choose from 3 possible answers, including: (a) None, (b) Yes, only once, (c) Yes, twice or more. Following previous practices (Wang et al., 2023; Yue et al., 2022), we coded falls as a dichotomous variable, that is, None = 0, and others = 1, treating the occurrence of any fall as a salient adverse event. Supplementary analyses using the original categorical measure are reported in Appendix A.

#### 2.2.3. Mediator Variable

Social adaptation was evaluated using the Social Adaptation Scale developed by B. Chen (2008), a measure that has been previously validated and applied in studies involving Chinese older adults (W. C. Chen & Wang, 2024; Li & Yu, 2020). The scale consists of eight items measuring different aspects of social adaptation (e.g., willingness to participate in community work). Each item was evaluated using a 5-point Likert scale, and negative items were reverse scored. In the current study, the scale showed acceptable internal reliability, with a Cronbach’ s alpha of 0.71 at T1. The total score ranged from 8 to 40, with higher scores reflecting greater social adaptation ability among older adults.

#### 2.2.4. Control Variables

This study controlled for a comprehensive set of covariates, including demographic, family, and socioeconomic characteristics, and health status. The demographic factors are composed of gender, age and birth cohort groups. Family and socioeconomic characteristics included educational attainment, marital status, living arrangement, residential region, Hukou and work status. Health status included chronic conditions and pain. Chronic conditions were coded as 1 if the respondent reported at least one chronic disease (e.g., hypertension, diabetes) and 0 if no chronic disease was reported (Yu et al., 2025). Finally, all regression models included a set of year dummies indicating the corresponding survey years to capture year variations. The details for these covariates can be found in Table 1.

### 2.3. Statistical Analysis

This study used a random effects (RE) regression model to examine the longitudinal association between falls and depression. A key advantage of RE models is that this approach allowed us to estimate both within-person temporal changes and between-person differences simultaneously, while efficiently utilizing all available data and maintaining statistical power for time-invariant covariates in our longitudinal analysis. The general form of the model can be expressed as follows:

(1) $$Y_{it}=\beta_{0}+\beta_{1}X_{it}+Z_{it}\delta +\nu_{i}+\varepsilon_{it}$$

In this equation, Y_it_ denotes the depression score of individual i at time t. The β_1_ coefficients indicate falls experience (0-no fall, 1-fall). The vector Z_it_ represents a set of control variables. ν_i_ is the individual-specific, time-invariant error term, and in RE models, ν_i_ is not correlated with explanatory variables such as falls and age. ε_it_ is the idiosyncratic error term that varies across individuals and time. All covariates that varied over time were treated as time-varying and updated at each wave across all models, whereas time-invariant characteristics were absorbed into the individual-specific effects.

In addition, we assessed the mediating role of social adaptation using both the Baron and Kenny method (Baron & Kenny, 1986) and the bootstrap method. Finally, to statistically test for Hukou differences, we estimated separate models for each Hukou status (rural and urban) and also estimated an interaction model. All analyses were performed using Stata 17.

## 3. Results

### 3.1. Sample Characteristics

Table 1 presents the characteristics of the full sample (N = 31,526), as well as subsamples of falls (n = 2972) and non-falls (n = 28,554). Observations were approximately 57% female and 51% aged 60–69 years. Around 12.3% of the observations had completed high school education or higher. The mean depression score was 6.39, with an SD of 3.22. Notable differences were observed in the dependent variables. Specifically, respondents who reported falls had a higher mean depression score (7.29) compared with those who reported no falls (6.29; t = −16.08, *p* < 0.001), and this value also exceeded the overall sample mean (6.39, SD = 3.22).

### 3.2. Regression Results

Table 2 presents the results of a random-effects regression model on the relationship between falls and depression among older adults. Here, we present the results unadjusted (model 1) and adjusted (model 2) for comparison. The coefficient for falls decreases from Model 1 to Model 2 but remains statistically significant, indicating a robust association between falls and depressive symptoms. Specifically, after adjusting for gender, age, birth cohort, education attainment, marital status, living arrangement, Hukou, residence area, having paid employment, chronic category conditions, pain, and survey year, falls were significantly associated with elevated depression symptoms (β = 0.625, *p* < 0.001). As a robustness check, we re-estimated the models using the original categorical measure of falls (none, only once, and twice or more), and the results were substantively consistent with those reported in the main analyses (see Appendix A).

### 3.3. Mediating Effect of Social Adaptation

The mediating effect of social adaptation between fall and depression was performed using the three-step method, and bootstrap standard errors were obtained. Model 3 of Table 2 shows that falls significantly negatively predicted social adaptation (β = −0.789, *p* < 0.001). Model 4 of Table 2 shows that social adaptation had a significant negative effect on depression (β = −0.069, *p* < 0.001). Falls had a significant positive effect on depression (β = 0.570, *p* < 0.001). It is worth noting that the coefficient for falls in Model 4 is reduced compared to the corresponding coefficient reported in Model 2. Therefore, it can be preliminarily determined that social adaptation plays a mediating role between falls and depression in older adults.

In addition, the results based on bootstrap mediation analysis showed that fall had a significant indirect effect on depression through social adaptation (β = 0.056, 95%CI = [0.037, 0.075], *p* < 0.001). Among them, the indirect path accounted for 8.91% of the total effect. Although the proportion of the total effect mediated by social adaptation is modest, this finding highlights its contributory role within a broader set of psychosocial pathways linking falls to depressive symptoms. Therefore, the mediating role of social adaptation has been further confirmed. The mediation analysis should be interpreted with caution, as it is based on a stepwise regression framework and bootstrap inference and does not establish causal mediation.

### 3.4. Moderating Effect of Hukou

**Figure 3:**
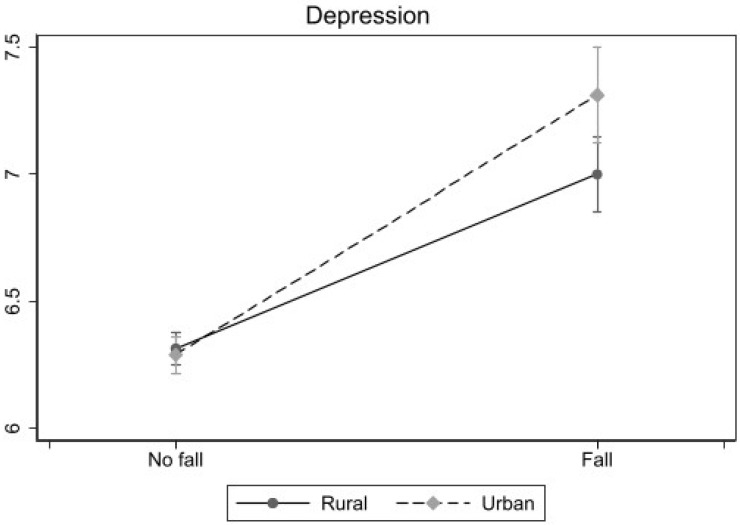
Predicted depression by fall and Hukou (rural, urban), based on models in Table 3, hold all other covariates at their means.

Table 3 illustrates whether Hukou moderates the relationship between falls and depression. Initially, we assessed the heterogeneity of this relationship across the two groups by employing separate sub-samples for rural and urban samples. Subsequently, we further examined the differential impact of this relationship between the two groups by incorporating an interaction term between falls and Hukou into our model. The results from both approaches consistently indicated that falls exert a more pronounced influence on depression among urban elderly individuals as compared to their rural counterparts. Finally, based on the results of the sub-samples in Table 3, Figure 3 was drawn to better present the differences in the relationship between falls and depression among urban and rural older adults.

## 4. Discussion

Drawing on four waves of longitudinal data from the China Longitudinal Aging Social Survey (CLASS), this study explored the association between falls and depression among older adults in China and its underlying mechanisms. The main findings can be summarized in three aspects: First, falls significantly exacerbate depression in older adults. Second, social adaptation mediates the relationship between falls and depression. Third, Hukou moderates this relationship, such that the negative impact of falls on depression is significantly stronger among urban older adults compared to their rural counterparts. These findings provide new empirical evidence for understanding the complex pathways through which falls affect the mental health of older adults.

This study found a positive association between falls and depression, which is consistent with prior research. Specifically, this finding aligns with a prior cross-sectional study conducted in China, which demonstrated that experiencing a fall in the past year was associated with increased depressive symptoms (Yang et al., 2023). More importantly, this study provides empirical support for the fear-avoidance model among Chinese older adults. This theoretical framework suggests that catastrophic interpretations of pain or injury can trigger fear responses, leading individuals to avoid social activities, which ultimately contributes to mental health deterioration (Peeters et al., 2020). In the context of this study, many elderly people interpret the catastrophizing of falls as a symbol of their own functional decline (“I can’t do it anymore”), thereby fostering a deep fear of falling again and gradually avoiding going out and social activities (Dashner et al., 2024). This kind of avoidance behavior not only accelerates the deterioration of their physical functions but also intensifies their sense of social isolation, thereby directly inducing or exacerbating depressive symptoms (Zhang et al., 2021).

A second key finding of this study is the mediating role of social adaptation. The results show that falls significantly weaken the social adaptation of the elderly, and a lower level of social adaptation leads to more severe depression. The mediating path of social adaptation accounts for 8.91% of the total effect. While this mediating effect is small to moderate in magnitude (Baron & Kenny, 1986), it represents a statistically significant pathway through which falls influence depression, highlighting social adaptation as one of multiple mechanisms that contribute to this relationship. This discovery reveals the intrinsic mechanism by which falls affect depression: falls may lead to a decline in the social participation and adaptability of the elderly (Yue et al., 2022; Zhang et al., 2021) as well as make it difficult for them to adapt to the changes in social roles and lifestyles brought about by changes in physical functions. This adverse social adaptation exacerbates depression. In recent years, social adaptation, as a core dimension for achieving active aging (Zeng & He, 2024), has been widely proven to play a crucial role in maintaining the mental health of the elderly (W. C. Chen & Wang, 2024; Zeng & He, 2024). Therefore, enhancing the social adaptation of the elderly has become an effective intervention measure to alleviate the risk of depression after falls.

This study also found that the relationship between falls and depression differs by Hukou status. Compared with rural older adults, the negative impact of falls on depression is more pronounced among urban older adults. This disparity may reflect differences in social environments between urban and rural areas. On the one hand, urban living environments (such as high-rise residences and more distant neighborhood relations) may be associated with reduced social participation and greater social isolation following a fall. On the other hand, the close social networks based on geographical proximity and kinship ties in rural areas may provide more immediate and effective informal social support for older adults after falls (Merril et al., 2006), thereby buffering the psychological impact brought by falls to a certain extent. This discovery suggests that when formulating targeted psychological intervention strategies after falls, the differences in social and cultural backgrounds between urban and rural areas must be fully considered.

Several limitations should be acknowledged. First, this study was unable to examine the long-term consequences of falls due to the focus of RE models on immediate effects. Future research can explore whether the effect between falls and depression follows a time pattern of attenuation, delay or repeated intensification, using dynamic structural equations. Second, the collection of falls relied on retrospective self-report, which can be affected by memory fading, cognitive decline, and social expectations, resulting in missed or false reporting. Future studies could improve the accuracy of fall measurement using smart wearable devices or hospital records. Third, although our study adopted a longitudinal design, the possibility of reverse causation cannot be completely excluded. For example, depression triggers slowness or medication side effects that lead to falls (Huang et al., 2025). Future research can utilize instrumental variables or individual fixed-effect cross-lagged panel models to enhance causal inference. In addition, to satisfy the longitudinal design, respondents observed in only a single wave were excluded, which may introduce selection bias and limit the generalizability of the findings to older adults with repeated survey participation. Finally, although mediation analyses were conducted to explore potential pathways linking falls and depressive symptoms, the mediator and outcome were measured within the same survey wave. As a result, the temporal ordering among falls, social adaptation, and depression cannot be firmly established, and the mediation findings should be interpreted as associational rather than causal. Moreover, the potential involvement of multiple mediating pathways-such as social participation and social support to falls could not be simultaneously examined in the present study and warrants further investigation. Future studies using cross-lagged panel models would allow for a more rigorous examination of causal mediation processes.

This study has important policy implications. First, given the substantial impact of falls on depression, fall prevention should be central to mental health promotion for older adults. Policymakers should prioritize evidence-based interventions, such as community-based fall risk assessments, Tai Chi and balance training programs, and home safety modifications (Aleixo, 2024; Sherrington et al., 2019). Second, since social adaptation mediates the fall-depression relationship, interventions should extend beyond physical prevention to adopt an integrated bio-psycho-social approach. Community health services should incorporate psychosocial support modules that enhance social adaptability through group activities, skills training, and life planning counseling, helping older adults—especially fall survivors—maintain social connections and self-worth. Finally, given significant urban-rural disparities, policies should be tailored accordingly. Urban interventions should focus on reducing social isolation and facilitating social network reconstruction, while rural interventions should strengthen existing kinship and neighborhood support systems by integrating mental health services into village governance and mutual aid networks. Such differentiated strategies can optimize resource allocation and effectively mitigate the fall-to-depression pathway, ultimately enhancing well-being among older adults.

In conclusion, this study enhances our understanding of the association between falls and depression. Falls can not only directly aggravate depression in older adults, but also indirectly increase depression by weakening social adaptation. Furthermore, compared with the rural elderly, the negative effect of falls on depression is more prominent in the urban elderly. Taken together, these findings suggest that preventing falls can not only alleviate depression but is also likely to become an important path to promoting healthy aging.

## Figures and Tables

**Table 1:** Descriptive statistics: Total sample and subsample.

	Total	Fall		*t*-Test/chi-Test (df)
		No	Yes	
	N = 31,526	N = 28,554	N = 2972	
Depression [0–18]	6.39 ± 3.22	6.29 ± 3.19	7.29 ± 3.32	−16.08 *** (31,524)
Gender				18.90 *** (1)
Male	51.5	51.9	47.7	
Female	48.5	48.1	52.3	
Age				157.56 *** (2)
60–69	51.0	52	41.4	
70–79	36.2	35.8	40.1	
≥80	12.8	12.2	18.5	
Birth cohort				252.97 *** (4)
Before 1935	5.9	5.6	9.1	
1935–1939	10.0	9.5	14.3	
1940–1944	16.1	15.7	19.8	
1945–1949	23.9	23.8	24.9	
1950 and after	44.1	45.4	32.0	
Educational attainment				211.20 *** (3)
Illiterate	22.7	21.7	33.0	
Primary	40.7	40.9	38.1	
Junior high	24.3	24.9	18.6	
High school and above	12.3	12.6	10.3	
Marital status				136.46 *** (2)
Married	72.8	73.7	63.8	
Widowed	25.7	24.8	34.6	
Divorced or never married	1.5	1.5	1.6	
Living arrangement				178.35 *** (4)
Living alone	11.4	11.3	12.6	
Living with only a partner	51.0	52.1	41.2	
Living with a partner and children	19.9	19.8	21.0	
Living with children	15.6	14.9	22.1	
Other living arrangements	2.0	1.9	3.2	
Hukou				89.10 *** (1)
Rural	56.6	55.8	64.8	
Urban	43.4	44.2	35.2	
Residence area				121.35 *** (4)
Downtown	36.4	37	30.1	
Suburb	9.8	10.1	7.6	
Combined urban-rural area	7.4	7.4	8.0	
Township	4.3	4.3	3.3	
Village	42.1	41.2	50.9	
Having paid employment				3.74 + (1)
No	78.6	78.4	79.9	
Yes	21.4	21.6	20.1	
Chronic category conditions				310.85 *** (1)
No	30.8	32.2	16.6	
Yes	69.2	67.8	83.4	
Pain				1100.00 *** (1)
No	57.3	60.3	28.5	
Yes	42.7	39.7	71.5	
Social adaptation	23.18 ± 6.20	23.30 ± 6.08	22.02 ± 7.10	10.80 *** (31,524)
Year				315.56 *** (3)
2014	9.9	9.0	19.1	
2016	28.6	28.8	27.3	
2018	33.4	33.7	30.8	
2020	28.0	28.6	22.8	

+ *p* < 0.1, *** *p* < 0.001.

**Table 2:** Baseline regression and mediating effect test.

	Model 1	Model 2	Model 3	Model 4
	Depression	Depression	Social Adaptation	Depression
Falls	0.801 ***	0.625 ***	−0.789 ***	0.570 ***
	(0.062)	(0.061)	(0.119)	(0.060)
Social adaptation				−0.069 ***
				(0.003)
Gender (Female = 1)		0.010	−0.429 ***	−0.020
		(0.045)	(0.090)	(0.045)
Age (ref. 60–69)				
70–79		−0.086	−0.118	−0.095
		(0.062)	(0.122)	(0.062)
≥80		−0.009	0.068	−0.005
		(0.118)	(0.230)	(0.117)
Birth cohort (ref. Before, 1935)				
1935–1939		−0.204 +	1.181 ***	−0.124
		(0.114)	(0.227)	(0.113)
1940–1944		−0.255 +	1.299 ***	−0.166
		(0.134)	(0.265)	(0.133)
1945–1949		−0.354 *	1.619 ***	−0.244 +
		(0.139)	(0.274)	(0.138)
1950 and after		−0.501 ***	1.777 ***	−0.380 *
		(0.151)	(0.297)	(0.150)
Educational attainment (ref. Illiterate)				
Primary		−0.217 ***	0.741 ***	−0.167 **
		(0.053)	(0.105)	(0.052)
Junior high		−0.595 ***	1.286 ***	−0.507 ***
		(0.064)	(0.126)	(0.063)
High school and above		−0.997 ***	2.363 ***	−0.834 ***
		(0.077)	(0.152)	(0.076)
Marital status (ref. Married)				
Widowed		0.257 **	0.013	0.259 **
		(0.079)	(0.156)	(0.079)
Divorced or never married		0.129	−0.528	0.093
		(0.179)	(0.355)	(0.178)
Living arrangement (ref. Living alone)				
Living with only a partner		−0.703 ***	0.211	−0.689 ***
		(0.087)	(0.171)	(0.086)
Living with a partner and children		−0.831 ***	0.161	−0.819 ***
		(0.092)	(0.181)	(0.091)
Living with children		−0.526 ***	0.010	−0.525 ***
		(0.072)	(0.142)	(0.072)
Other living arrangements		−0.273 *	−0.211	−0.289 *
		(0.137)	(0.268)	(0.135)
Hukou (Urban = 1)		0.015	1.041 ***	0.087 +
		(0.052)	(0.102)	(0.052)
Residence area (ref. Downtown)				
Suburb		−0.069	0.023	−0.067
		(0.066)	(0.129)	(0.065)
Combined urban-rural area		−0.005	−0.944 ***	−0.070
		(0.076)	(0.150)	(0.076)
Township		0.409 ***	0.753 ***	0.461 ***
		(0.094)	(0.185)	(0.093)
Village		0.513 ***	0.058	0.516 ***
		(0.059)	(0.117)	(0.059)
Having paid employment		−0.287 ***	0.154 +	−0.277 ***
		(0.046)	(0.090)	(0.045)
Chronic conditions		0.457 ***	−0.048	0.453 ***
		(0.042)	(0.082)	(0.041)
Pain		0.740 ***	−0.662 ***	0.695 ***
		(0.038)	(0.074)	(0.037)
Year (ref. 2014)				
2016		1.832 ***	−2.433 ***	1.665 ***
		(0.058)	(0.112)	(0.058)
2018		1.980 ***	−2.132 ***	1.833 ***
		(0.059)	(0.115)	(0.059)
2020		2.127 ***	−2.067 ***	1.985 ***
		(0.064)	(0.124)	(0.063)
Constant	6.326 ***	5.018 ***	22.813 ***	6.591 ***
	(0.024)	(0.176)	(0.347)	(0.187)
**Bootstrap**	**β**	**95% CI**		**% of total**
Indirect effect	0.056 ***	[0.037, 0.075]		8.91%
Direct effect	0.570 ***	[0.452, 0.688]		
Total effect	0.626 ***	[0.508, 0.744]		
Observations	31,526	31,526	31,526	31,526

Standard errors in parentheses. + *p* < 0.1, * *p* < 0.05, ** *p* < 0.01, *** *p* < 0.001.

**Table 3:** Random-effect regression models of depression on falls and Hukou.

	Model 1	Model 2	Model 3
	Depression		
	Urban	Rural	Total
Falls	0.723 ***	0.489 ***	0.505 ***
	(0.100)	(0.077)	(0.076)
Hukou (Urban = 1)			−0.011
			(0.053)
Urban # Falls			0.320 **
			(0.123)
Observations	13,681	17,845	31,526

Standard errors in parentheses. ** *p* < 0.01, *** *p* < 0.001. All models controlled for gender, age, birth cohort, educational attainment, marital status, living arrangement, Hukou, residence area, paid employment status, chronic conditions, pain, and survey year.

## Data Availability

The data that support the findings of this study are available from CLASS project site, subject to registration and application process. Further details can be found at http://class.ruc.edu.cn. We agreed to share the data from this study if we obtain permission from the CLASS project.

## Appendix A. Robustness Check Using the Categorical Measure of Falls

	**Model 1**	**Model 2**	**Model 3**	**Model 4**
	**Depression**	**Depression**	**Social Adaptation**	**Depression**
Falls (ref. none)				
Only once	0.794 ***	0.550 ***	−0.536 ***	0.513 ***
	(0.072)	(0.070)	(0.137)	(0.069)
Twice or more	0.819 ***	0.823 ***	−1.460 ***	0.722 ***
	(0.111)	(0.109)	(0.214)	(0.108)
Social adaptation				−0.069 ***
				(0.003)
Control variable		Controlled	Controlled	Controlled
Constant	6.326 ***	5.007 ***	22.851 ***	6.580 ***
	(0.024)	(0.177)	(0.347)	(0.187)
Observations	31526	31526	31526	31526
Standard errors in parentheses. *** *p* < 0.001.				
